# 血液专科重症单元危重症患者诊疗和管理中国专家共识（2025年版）

**DOI:** 10.3760/cma.j.cn121090-20241206-00542

**Published:** 2025-01

**Authors:** 中华医学会血液学分会

## Abstract

近年来，恶性血液病患者的生存期明显延长，血液恶性肿瘤患者严重并发症的发生率亦相应升高，且病情常变化急骤。设立血液专科重症单元（HCU）对于早期识别、集中监护式管理血液危重症患者，提高血液科诊治水平、改善患者预后、降低医患纠纷风险具有重要临床意义。参考综合重症监护病房（ICU）和血液科建设指南，结合国内临床HCU运行经验，由国内多个血液临床诊疗中心参与的专家组完成本共识。本共识在HCU建立的必要性和重要性、基本配置标准、收治标准、管理制度、原发病治疗原则及诊疗策略等方面给出了具体意见，并建议具备相应条件的血液中心积极建立HCU，以期进一步降低血液重症的死亡率。

## Abstract (en)

Recently, survival rates for patients with hematological malignancies（HMs）have improved, but severe complications have also risen accordingly, and can escalate quickly. Establishing a hematology intensive care unit（HCU）is crucial for early detection and centralized monitoring of critically ill hematological patients, as well as the enhancement of diagnosis, treatment and prognosis and minimizing medical disputes. Based on the guidelines of intensive care unit and hematology department, combined with clinical operation experience of domestic HCU, this consensus is participated by several leading hematology centers in China. It outlines the necessity, configuration, admission standards, management, treatment principles, and strategies of HCU. An expert agreement on managing critically ill HCU patients was achieved. It is suggested that the blood centers with eligible critieria should actively establish HCU, aiming to further lowering the mortality rate of critically ill hematology patients.

近年来，恶性血液病（HM）患者的生存预后和生活质量明显改善[1]–[2]，主要与精准诊疗技术的推广应用、高强度化疗的临床使用、靶向治疗的快速发展、造血干细胞移植理论和临床的进步、CAR-T细胞治疗与免疫治疗的不断更新及抗感染等辅助支持治疗的完善有关。但HM患者中老年患者比例逐渐增加，伴随着新型靶向及免疫治疗的积极推进和应用，相关合并症更复杂，危重并发症发病率明显上升。而且，血液危重症患者在住院期间存在病情变化急骤、进展迅猛、预后差的特点，诊断、治疗等方面都具有较强的专科特征，因此综合重症监护病房（intensive care unit，ICU）和血液科专科的诊治经验，设立血液专科重症单元（hematology intensive care unit, HCU），早期、及时转入急症和危重患者，集中诊断、治疗和护理，加强生命体征监测和支持，有利于改善此类患者的预后，提高血液危重患者的诊治水平，降低医患纠纷风险。鉴于目前国内外尚缺乏全面、系统的针对血液科危重症患者综合管理和诊疗的专家意见，专家组参考ICU和血液科建设指南，结合国内临床HCU一线运行经验撰写本共识，以期为血液科危重患者住院管理中的一些特征和关键事项提出可行的建议。

一、HCU设置的重要性

HM多起病急骤，可累及全身多个组织器官，病情复杂不易诊断，且部分类型进展迅速危及生命。约13.9％的HM患者在确诊一年内需要转入ICU进行高级生命支持及相关治疗，其中惰性淋巴瘤重症患者需转入ICU的比例为7.3％，而血液科需住院治疗的最主要病种急性白血病中重症患者的比例则高达22.5％[3]。文献报道，危重症HM患者早期（出现危重症状或诱导化疗前）转入ICU的生存率约为79％，而在较晚阶段紧急转入ICU的生存率则下降至65％[4]–[5]。血栓性血小板减少性紫癜（TTP）等疾病具有很强的血液专科特性，起病急，早期误诊和漏诊率较高，如重症TTP常合并神志障碍、肾衰竭、重症感染等严重并发症，在给予高级生命支持的同时，也需要针对原发血液病给予及时、准确的治疗。重型噬血细胞综合征（HLH）病情危重、进展迅猛，合并细胞因子风暴和过度炎症反应综合征，容易导致肝衰竭、弥散性血管内凝血（DIC）等多器官功能衰竭，临床死亡率高达40％，如不能及时给予专科处理并积极控制相关并发症，预后极差。HM患者由于免疫缺陷发生院内获得性肺炎（HAP）、侵袭性真菌病、病毒感染等的概率极高，且血流感染的发生率为11％～38％，部分重症患者可迅速进展为脓毒败血症休克[6]，病死率超过40％[7]–[8]，72 h内有效经验性抗生素治疗可显著降低相关死亡率。部分HM易并发肺炎、败血症、白血病浸润或肺部移植物抗宿主病等，导致急性呼吸衰竭（ARF），相关死亡率为30％～70％，这也是HM患者转入HCU的最常见原因[9]–[10]。一项队列研究报道，转入ICU的HM患者约51％需要使用去甲肾上腺素等血管活性药物，26％的患者需要血液透析和超滤，48％需要气管插管行有创机械通气（IMV）[2]–[3],[5],[11]，死亡率极高。

鉴于HM起病急骤、进展迅猛的专科特点，尽早发现、集中而有效的治疗有利于降低这类患者的早期死亡率，需要管床医师既具有极强的血液专科知识储备，又同时具备处理危重并发症的综合能力，能及时给予经验性及针对性治疗，减少因并发症导致的死亡风险，为原发病治疗提供更多机会。如果此类患者能在HCU进行早期监测并及时接受生命支持治疗，有利于提高救治成功率，或延长生存时间。因此，建立HCU对于降低血液科危重症患者死亡率、优化医疗资源分配、提高HM的整体救治效率均具有重大意义。通过集中管理危重患者，有助于临床医师探索此类患者的疾病发展规律，制定规范的诊疗指南，培养更多专业的血液重症医护人员，落实国家大力发展专科ICU的精神，推动重症医学专科队伍建设。

为积极有效救治血液危重症患者、及时把握血液专科诊疗时机，2011年底华中科技大学同济医学院附属协和医院血液病学研究所率先成立了HCU，目前哈尔滨医科大学附属第一医院、中国医学科学院血液病医院、苏州大学附属第一医院等多家医院陆续成立HCU，为疑难危重血液病患者提供全面、综合的专科诊疗和护理，努力提高成功救治率。湖北省内荆州、襄阳、宜昌等地也已成立或在积极筹建HCU。

HCU改善了部分血液重症患者无法及时转入ICU救治的弊端，为此类患者提供了及时有效的救治；有并发危重症临床特征的HM患者，在HCU接受早期集中管理后能有效降低危重症发生率，从而降低此类危重血液患者的死亡率。HCU医师能在治疗严重并发症的同时治疗原发血液病，体现专科疾病救治方面的优势。

二、HCU的定义和配置条件

HCU是指同时掌握血液专科和急救、重症医学等诊疗经验的医疗、护理团队，基于多学科协作的诊疗理念，利用先进的监测设备、诊断技术和高级生命支持措施，对血液病重症患者实施专科化、目标化精准诊疗的医疗单元，应该具备符合条件的医护人员、独立的场所及必要的设施和设备。基于HCU的不同运行模式，专家组提出了两种HCU配置条件以供参考。一种是ICU模式，由高级以上职称的血液专科医师与ICU专科医师于血液重症病房协作管理患者，并参考《中国重症加强治疗病房（ICU）建设与管理指南》（2006）[12]进行环境及设备配置，运行模式与ICU基本一致，此处不予以详细阐述。而基于血液危重患者的专科特点，专家组也对另一种运行模式达成共识，即pre-ICU模式，由经过ICU培训并获得相应资质的血液专科医师主导管理患者，ICU作为会诊科室辅助管理。建议如下：

1. 人员配置：①三级医师梯队，有明确的分工和侧重：主任医师由有长期HCU工作经验的主任医师担任，负责制订疑难危重患者的诊治方案；主治医师由临床经验丰富、善于沟通的中年骨干医师担任，负责协调和安排外院、急诊和科内患者的收治、转出等；另外配置1位高年资住院医师和1位住院医师，在上级医师指导下完成患者的具体医疗工作，包括临床基本操作、安排常规检查和查阅检查报告、病情变化的整理和汇报等。②护理团队，护理人员与床位数配比建议≥2∶1，需掌握分管患者的病情和监测重点、基本护理技能、床旁吸痰、无菌操作，识别心电监护的异常指标，掌握急救仪器（人工呼吸机、监护仪、心脏除颤仪等）的使用方法、仪器报警和故障的排除方法并了解其使用目的，熟悉心脏骤停等紧急情况的处理流程。另外需配置主管护师负责PICC置管和输液港植入、股静脉置管、白细胞单采、血小板单采、血浆置换等，经鼻高流量氧疗和无创呼吸机的使用等。

2. 环境配置：建筑、环境符合规范，利于危重患者的救治和医院感染的监控工作：①中央监护站可24 h连续监测患者的生命体征，紧急报警的声音和灯光应同时传达至中心护士站、值班室；②患者床单位至少15 m^2^，足够安放必需的仪器设备，为必要操作留有足够的活动空间；③具备良好的通风、采光、照明条件，配置空气净化和紫外消毒设备、层流装置；④备有隔离（应急）床位，配备不间断电源系统和可自控应急供气、供电装置。

3. 仪器设备配置：能够敏感、准确地提供实时的监护数据，根据各级医院的具体情况，建议参考如下配置方案：①必备设备：带有心电图、呼吸频率、血压、血氧饱和度模式的联网多功能监护仪/中央监护站，床边电源，中心氧供，负压吸引系统和多功能气垫床，输液泵，注射泵等；②公用设备：复苏呼吸气囊和心肺复苏抢救装备车，心电图机和除颤仪，无创大呼吸机、简易呼吸机、经鼻高流量氧疗呼吸机，血气分析仪，血浆置换和血细胞单采去除设备，电子降温毯、等离子消毒机等；③选配设备：血液透析仪、心脏起搏设备、纤维支气管镜、电子胃镜等；④院内建立相关多学科团队（MDT）提供咨询和帮助：本科室或院内能及时提供床旁血液过滤装置、血液净化装置、移动式X线诊断装置、超声诊断装置及紧急麻醉科会诊行气管内插管等，部分危重患者及时转入综合ICU治疗；有条件的单位建议与院内呼吸治疗师、电生理技师、康复理疗师、临床药师、营养师等保持积极沟通与联系。

三、HCU的收治对象

HCU不是大型综合医院ICU分支，主要对病危、病重和急症的HM患者进行ICU的前置综合收治。与综合ICU相比，HCU有其专科特点：①血液专科危重症多，对血液原发病及治疗相关并发症的处理较综合ICU更具血液专业性；②血液病患者往往伴免疫缺陷、粒细胞缺乏、重症感染等并发症，血液科医师具备有专科特色的感染诊断和治疗经验。

HCU的收治对象主要针对起病急、进展迅速、预后差的血液病危重患者，或者在治疗过程中出现急剧病情变化的患者，及部分需要ICU紧急救治但由于床位紧张或专科疾病特点受限等特殊原因无法收治于ICU的患者。其生命体征多不平稳，合并内环境紊乱，因此，同样需要24 h心电监测和高级生命支持及MDT协作诊疗模式。具体收治对象包括如下几大类：①高度疑诊或确诊为进展迅速的高致死性血液专科疾病患者，包括血栓性血小板减少性紫癜、HLH、高白细胞急性白血病（HAL）等；②血液病合并严重并发症患者，包括感染性休克、脓毒症、DIC、严重消化道出血、累及中枢神经系统或颅内出血等；③血液病合并重要脏器功能不全患者，包括呼吸衰竭、心力衰竭、肾功能衰竭、肝功能衰竭等；④造血干细胞移植或新型免疫治疗后出现严重并发症患者，如超急性移植物抗宿主病（GVHD）、3～4级细胞因子释放综合征（CRS）或免疫效应细胞相关神经毒性综合征（ICANS）、3～4级免疫检查点抑制剂相关间质性肺疾病（ICIs-ILD）等；⑤其他危重症，需要生命支持及24 h监护的患者。患者转入HCU后，予告病危或病重，行全天心电监护连续监测生命体征，出现异常可以及时处理；根据病情予呼吸机辅助通气、血浆置换、血细胞单采、连续性肾脏替代治疗（CRRT）等支持治疗。

四、HCU的管理制度

为切实提升HCU的临床诊治水平，提高血液危重症患者的救治成功率，保持专科优势，促进学科发展，除了基本核心医疗制度以外，应根据专科疾病特点补充相应的管理制度，主要包括：

1. 转入与转出制度：收治的急诊或会诊患者需排除其他专科疾病和危及患者生命的其他专科情况，符合HCU收治标准，并请血液科医师会诊确定后收治；血液科住院患者病情危重符合HCU收治标准，各医疗组负责医师需与HCU医师沟通，并与患者及家属谈话，签署HCU转入同意书（同意或拒绝）。患者病情变化时是否需要转入HCU需要管床医师和HCU医师根据原发病的转归情况、转诊前的治疗经过、患者的生命体征和意愿等综合评估，在讨论和转诊时应明确说明并记录以下信息：①原发病转归情况、治疗计划和预后；②既往与患者商定的任何治疗限制；③特殊输血要求；④隔离需要等。

转出标准应根据患者转入HCU的原因和当时的病情状态连续动态监测和评价：①生命体征平稳，不再需要连续动态监测和HCU治疗的患者，可转入专科普通病房继续治疗或出院后定期门诊随访；②生理状态进行性恶化，积极的干预性治疗疗效不佳，尽早明确重症监护和器官支持等对症治疗对原发病和生存预后的作用，避免过度无效治疗；必要时与患者及其直系亲属等进行有效沟通，适时转为临终管理和姑息治疗。

超过20％的危重患者转运途中会发生不良事件，主要原因是设备问题和人为因素，因此院内和院际转运过程应遵循ICS危重成人转运指南[13]–[14]，优化书面规则和程序，深入协调和沟通，定期培训，提高转运团队的专业素养，定期检查监护和抢救设备的工作状态等。严格落实专科医师查房制度、危重患者床边交接班制度、疑难病例会诊、多学科协作诊疗并对危重病例和死亡病例进行讨论等，确保HCU危重症患者优先获得连贯医疗救治。

2. HCU留陪制度和访视制度：ICU的患者病情危重，免疫力低下，加上侵入性操作，院内感染发生的风险极高，病房常采取封闭式、家属无陪护的管理模式；但长时间与家属分离，孤独感和消极情绪剧增，病情信息的缺乏、疾病或死亡的威胁等现实问题，容易使部分患者出现焦虑、抑郁、认知障碍等ICU综合征[15]。随着生理-心理-社会医学模式的建立，以患者家庭为中心的探视制度开始广泛应用于新生儿科、儿科和肿瘤患者[16]–[17]。建议根据运行情况选择采取ICU限制式探视模式（每日探视人数不超过2人，每日1次，固定探视时间）或pre-ICU灵活的家庭探视模式（固定留陪1人，根据患者病情或家属需求增加其他家属成员的间断短时间探视，有条件者可利用电子设备进行探视）。留陪需为家属提供健康知识的培训和教育，指导家属参与到患者的共同决策和护理中，有利于患者疾病的康复，提高家庭总体对医护的满意度；同时应当增加仪器消毒和空气消毒频率等。

3. HCU日常工作流程：①对于紧急转入的危重症患者，设计简易的临床资料收集表格，主要包括患者的一般信息，血管活性药物、氧气等生命支持措施，原发病状态，抗生素使用经过，各种感染相关指标、样本（血液、痰液、引流液、分泌物等）病原微生物培养和基因测序检测信息、可疑感染部位评估（口咽、尿道口、肛门、皮肤破损处等），影像学检查和其他重要辅助检查结果等，及时、全面、直观地对危重患者的病情进行综合评价，根据病情变化作出诊治方案的调整。②对于行专科化疗的HM患者，设计全面的临床资料随访表格，主要包括患者的一般信息和既往病史，原发病的危险分层和预后评估，初诊时的血象特点和主要器官功能状态，既往化疗方案和化疗后评估结果，化疗过程中和化疗后发生的严重不良事件等，综合、全面评估患者对化疗方案的耐受情况和原发病转归情况，慎重决定后续的诊疗方案。③定期（每周2次以上）筛查发热患者常见呼吸道病原体咽拭子、碳青霉烯耐药肠杆菌科细菌（CRE）肛拭子，肛拭子CRE阳性患者需完善咽拭子、尿道口、皮肤破损处等CRE的筛查。

五、血液专科重症患者疾病状态和功能评估

（一）基本生命体征评估与监测

重点关注患者有无神志淡漠、咽痛、胸痛、胸闷、腹痛、腹泻等预警症状，对危重症患者实施量化评估（包括SIRS四项标准、SOFA、qSOFA[18]及APACHE Ⅱ[19]评分等），全面掌握患者的整体状况；根据各项基本指标迅速评估患者各个器官的功能障碍情况：①收缩压<90 mmHg（1 mmHg＝0.133 kPa），平均动脉压<70 mm Hg；心脏指数（CI）<2.2 L·min^−1^·m^−2^提示休克或心力衰竭；②氧合指数（PaO_2_/FiO_2_）<300 mmHg，血清乳酸>3 mmol/L提示低氧血症或急性呼吸窘迫综合征（ARDS）；③急性少尿、尿量低于0.5 ml·kg^−1^·h^−1^，持续2 h以上，血肌酐增加≥44.2 µmol/L提示急性肾损伤；④总胆红素>70 µmol/L，肝酶异常升高或胆酶分离提示肝功能衰竭；⑤PLT<10×10^9^/L，凝血指标异常，包括APTT>60 s，纤维蛋白原（FIB）<1.0 g/L或国际标准化比值（INR）>1.5。密切监测患者重点指标及病情变化，及时调整治疗方案，有效维持重症患者的基本生命体征。

（二）专科查体

血液系统专科查体：包括意识状态（瞳孔大小及瞳孔对光反射、病理征）、生命体征、皮肤和巩膜色泽、皮肤、黏膜是否有瘀血瘀斑、皮下及内脏出血、鼻出血、牙龈出血等，胸骨压痛、心肺听诊、肝脾肿大、双下肢水肿等情况。

（三）感染分层

推荐根据癌症患者支持治疗的多国协作组织（MASCC）评分系统[20]对患者进行感染分层，风险评分≥21分提示患者发生并发症及死亡的风险可能较低。同时评估患者以下情况：是否高龄，原发病是否缓解，是否有严重中性粒细胞缺乏或预期粒细胞缺乏期持续7 d以上，有无广泛严重的黏膜损伤，是否合并低血压，口腔或胃肠道黏膜炎，腹痛、恶心、呕吐、腹泻等胃肠道症状，新发的神经系统症状或精神状况，血管内导管感染，新发的肺部浸润或低氧血症，肝功能不全（谷丙转氨酶/谷草转氨酶>5倍上限），肾功能不全（肌酐清除率<30 ml/min）。符合以上1项标准即为高危患者。

（四）静脉血栓和出血高风险评估

静脉血栓栓塞症（VTE）是危重患者常见且后果严重的并发症，包括深静脉血栓（DVT）和肺栓塞（PE）；高危因素包括长期卧床、高龄、中心静脉留置导管、血液高凝状态、重症感染等[21]。内科住院患者由护士根据Padua评分表评估VTE风险，其中评分≥4分的高危患者需根据具体病情评估血栓风险[22]。推荐在患者转入HCU时进行DIC评分，确诊DIC者每日监测动态指标和评估出血风险，按照《弥散性血管内凝血诊断中国专家共识（2017年版）》[23]予以输血、抗凝等对症支持治疗。疑诊DVT的高危患者需动态监测D-二聚体等指标变化，必要时完善超声、CT静脉成像等检查；强调早期预防、全程治疗：物理预防可以通过间歇充气加压泵和加压弹力袜增加下肢静脉血流回流，减少静脉血流淤滞；药物预防有低分子肝素、肝素、新型口服抗凝药物等；一旦确诊，无绝对禁忌证的高危患者要尽早开始抗凝预防，在多学科会诊基础上制定个体化、综合干预方案。

六、血液重症患者专科疾病处理原则

对于危重症HM，原发病治疗是一项慎重而艰难的选择。目前没有可靠的路径或指南，本文提供的专家建议仅供参考。

（一）患者是否适合进行原发病治疗

建议结合患者的一般情况、原发病治疗阶段、家属治疗意愿等进行综合评估，予以适当分类。

1. 不建议开始或继续原发病治疗的患者主要包括：①预期生存期不足1周的患者；②多次复发或疾病终末期患者；③治疗方案很可能加重危重症患者的病情，如重症感染时应用免疫抑制药物、肝肾功能衰竭时应用细胞毒性药物等；④病情危重，包括症状体征或相关辅助检查结果等尚在加重或提示病情进展；⑤未取得其家属对原发病充分理解的患者。

2. 建议慎重进行原发病治疗的患者，包括：①原发病治疗过程中发生的危重症（如诱导化疗过程中因发生DIC、ARDS等转入HCU）不能排除与化疗相关的患者；②病情危重处于不稳定阶段的患者；③高龄患者（≥75岁）；④重要器官或系统功能处于完全失代偿期的患者。

3. 建议进行原发病治疗的患者，包括：①初诊HM，年龄≤60岁，发生危重症前合并症指数APACHEⅡ评分<20分且生理指标<4分或无严重器官功能不全的患者；②危重症病情稳定1周以上或好转3 d以上的患者；③治疗原发病的方案或药物机制可能减轻危重症的致病因素，如急性早幼粒细胞白血病（APL）诱导化疗针对DIC、CD20单抗治疗针对CD20阳性淋巴瘤合并HLH等；④家属充分理解配合，对原发病治疗持积极态度。

（二）HM原发病治疗方案选择建议

HM原发病治疗方案选择建议参照以下情况：

1. 按照疾病一般状况评价中的Unfit（不耐受）或Frail（脆弱）的治疗原则；

2. 选用安全性高、特异性好的药物如新型靶向治疗药物；

3. 对方案中的药物进行选择和剂量调整，进行桥接化疗，如弥漫大B细胞淋巴瘤（DLBCL）应用R-CHOP方案时可先去掉阿霉素和环磷酰胺，待病情好转时再行标准方案治疗。

七、血液专科重症患者重要并发症的管理

（一）重症感染

HM患者是发生重症感染的高危人群，由于原发性和继发性免疫功能缺陷（与化疗、放疗、造血干细胞移植等治疗相关），出现粒细胞缺乏伴发热、CRE感染、真菌感染、病毒感染等，极易迅速进展为脓毒性休克，重症感染的发生率和病死率明显升高。其特点包括：①院内感染常见，铜绿假单胞菌、鲍曼不动杆菌等耐药菌及非发酵菌比例增加[24]；②侵袭性真菌病（invasive fungal disease, IFD）比例增加；③多种病原体混合感染[25]。血液重症感染患者分离出的病原体：除常见超广谱β-内酰胺酶（ESBL）（+）的革兰氏阴性杆菌、耐甲氧西林金黄色葡萄球菌（MRSA）外，还有耐药不动杆菌、CRE、耐药铜绿假单胞菌、利奈唑胺中度敏感的肠球菌等[26]。其中碳青霉烯类抗生素的使用是导致铜绿假单胞菌、嗜麦芽窄食单胞菌多重耐药的风险因素。

基于以上HCU患者的特点，其抗感染治疗要点包括：①尽早抗感染治疗，“高阶梯”治疗[26]；②足量治疗：与非危重患者相比，危重患者的抗生素药物动力学有明显改变，因此有时要加量治疗（在参考共识和重要文献的情况下），制定更加个体化的剂量方案[27]；③注意不同药物的抗菌特性，根据感染部位及抗菌需求选择抗生素；④HCU患者通常合并多重耐药菌（MDRO）危险因素，应该覆盖常见耐药菌[25]；⑤对高危患者进行真菌预防及经验性抗真菌治疗[28]；⑥联合用药，对于脓毒症或脓毒症休克患者，推荐经验性使用可能覆盖所有病原体的抗菌药物[29]。

（二）DIC

急性白血病可在多种因素的作用下并发DIC，最常见于APL，也包括存在高白细胞症状及严重感染的患者，治疗要点包括：①治疗原发病和去除诱因是终止DIC病理过程最关键和根本的措施；②在干预原发病的前提下，同步进行凝血因子的补充与普通肝素、低分子量肝素等抗凝治疗（详见《弥散性血管内凝血诊断中国专家共识（2017年版）》[23]），从而阻止凝血过度活化、重建凝血-抗凝平衡、中断DIC病理过程；③替代治疗：主要使用新鲜冰冻血浆等血液制品、血小板悬液、FⅧ及凝血酶原复合物，以控制出血风险和临床活动性出血为目的；④其他治疗包括对症支持治疗、纤溶抑制药物治疗、糖皮质激素治疗。

（三）VTE

VTE的治疗和处理是一个复杂的过程，但主要以抗凝治疗为基础。DVT常见症状包括单侧肢体肿胀、疼痛等。PE可能表现为突发呼吸困难、胸痛、低氧血症等，尤其需要与其他重症表现相鉴别。①抗凝治疗是VTE治疗的核心。一旦诊断为VTE，应尽早进行抗凝治疗，以预防血栓的进一步蔓延和复发。②抗凝药物的选择：普通肝素适用于急性VTE的起始抗凝和持续给药，特别是静脉置管溶栓后的过渡治疗。低分子肝素推荐用于急性VTE的起始抗凝，尤其是大面积PE的起始治疗。直接口服抗凝药物（direct oral anticoagulant, DOAC）如利伐沙班、阿哌沙班等适用于VTE急性期和长期单药抗凝。③治疗性抗凝适用于所有已发生VTE的患者，无大的出血风险。对出血相关风险进行评估后，应该达到足够的治疗剂量和疗程。④评估和监测：在抗凝治疗过程中监测D-二聚体水平和出血情况，以及时调整治疗方案。定期进行超声和（或）CT检查，评估血栓的变化和治疗效果。有出血并发症时，应立即停止抗凝治疗，并进行必要的支持治疗和止血措施。

八、血液专科重症患者的重要对症和脏器支持治疗

（一）呼吸支持

血液病重症患者多合并重症感染，常伴呼吸功能障碍，气道分泌物排出不畅，重者发生低氧血症和呼吸衰竭。HM合并急性呼吸衰竭的氧疗方式包括单纯吸氧（鼻导管和面罩给氧）、经鼻高流量氧疗（high-fow nasal cannula oxygenation, HFNO）、无创机械通气（noninvasive mechanical ventilation, NIV）和气管插管行有创机械间歇指令通气（invasive mechanical ventilation, IMV）[9]。HFNO可以精确控制气体的温度、湿度和氧流量，降低气体对气道黏膜纤毛的刺激；可产生气道正压，显著改善患者的呼吸功能和氧合指数；无面部压迫感，方便进食、交流，提高患者的舒适度和耐受性[30]。

有研究表明，早期使用HFNO和NIV适用于PaO_2_/FiO_2_>200 mmHg或SpO_2_<90％，呼吸频率>25次/min的轻症低氧血症和可逆性病因患者，若NIV疗效欠佳或患者病情加重则需要尽早升级为IMV；对于神志异常、血流动力学不稳定、PaO_2_/FiO_2_<200 mmHg，呼吸频率>25次/min、ARDS及合并多脏器功能衰竭的患者则首选IMV[11]。进行呼吸支持时注意有无人机对抗，采取必要的镇痛镇静管理，避免发生呛咳或呃逆，积极治疗获得性肺炎，预防呼吸机相关性肺炎，强化气道管理。

（二）液体和血流动力学管理

液体管理对于危重症患者的救治极为重要，液体入量过多易导致患者循环血容量急剧增加，造成应激性心力衰竭；而入量不足则易导致血流动力学不稳定，组织缺血缺氧、代谢紊乱甚至休克等严重并发症[31]。高白细胞血症由于大量白血病细胞淤滞，易发生DIC、颅内出血、脑梗死、ARDS等危及生命的并发症，因此需要足量液体2 000～3 000 ml·m^−2^·d^−1^水化，5％碳酸氢钠80～100 ml·m^−2^·d^−1^碱化尿液。

对危重患者进行液体复苏时，早期诊断和纠正循环功能不足和组织灌注不良，及时调整输液的种类、总量和速度，对提高抢救成功率至关重要[32]。血流动力学监测包括：①动脉压、皮肤花斑评分和毛细血管再充盈时间（CRT）[33]；②无创血流动力学监测[34]–[35]，利用胸腔生物阻抗技术计算无创心输出量（CO）和心脏指数（CI），动态床边心脏超声、胸腹水超声可作为补充手段；③中心静脉压检测和肺动脉漂浮导管，有创性操作，有肺梗死、穿刺感染、导管血栓等风险，对设备和医师的技术水平要求高，在连续和早期监测中存在一定局限性。

（三）CRRT

CRRT是重症监护中器官支持的重要组成部分之一。一般来说，最常用于重度急性肾功能衰竭（ARF）和相关并发症［如重度代谢性酸中毒、尿毒症、重度电解质和代谢紊乱和（或）液体超负荷］，可应用于血流动力学不稳定和颅内高压患者。由于肿瘤溶解综合征（TLS）、使用大剂量化疗药物、严重感染及原发病造成的肾功能损伤等，HCU患者常出现药物难治性严重代谢性酸中毒、急性肺水肿、难治的严重高钾血症、其他电解质紊乱、顽固性液体超负荷伴器官功能障碍及进行性加重的ARF等，因此在合适时机启动CRRT可以改善这部分患者的预后，降低病死率[36]。对于感染性休克、低血压患者，在使用血管活性药物及适当补液维持血压的情况下，也可使用CRRT干预。而当凝血功能障碍或轻至中度活动性出血患者存在紧急CRRT指征时，仍可通过采取无肝素抗凝或枸橼酸局部抗凝等方式进行CRRT。

（四）镇痛镇静

合理的镇静镇痛可以控制患者焦虑、躁动和谵妄，减轻其应激反应，最大限度保留基本的反射、感觉和运动功能，减轻各器官代谢负担，促进患者机体器官功能的恢复。镇痛不足会导致患者氧耗增加，影响疾病康复和预后，而过度镇静会延迟苏醒时间，削弱自我保护反射，导致心力衰竭、内环境紊乱等严重并发症[37]。2016年，Vincent等[38]提出以患者为中心的舒适化镇痛镇静方案（eCASH），采用滴定式评估，维持合理的镇痛水平，减少镇静手段的使用，改善患者的自然睡眠状态，有利于患者的早期功能锻炼，以及与医护人员、家属的沟通。

（五）营养支持

HCU患者病情较危重，处于高分解代谢状态，部分患者存在恶心、呕吐等症状，不能进食或处于禁食状态，需早期给予合理的营养支持，补充足够的能量和营养素，维持生命和改善状况、缓解病情[39]–[40]。肠外营养是严重营养不良、肠内营养禁忌等患者唯一的营养支持方式，但会减弱胃肠蠕动和消化吸收功能，导致肠道细菌移位，影响肝功能以及引起胆汁淤积，诱发全身感染、导管感染；肠内营养是生理性营养给予途径，能刺激胃肠道分泌，维持胃黏膜完整和内脏血流稳定，降低应激性溃疡风险，预防细菌移位，且用药方便，不良反应少[41]。

在条件允许的情况下，建议请营养科会诊或配备营养师，营养原则强调早期启动、缓慢增加和重视补充蛋白质，建议入院48 h内即启动肠内营养，初期1～3 d可供给的目标能量建议40％～70％，随后增加到80％～100％。肠内营养联合肠外营养支持能提高患者的耐受力，保证营养供给满足需求，降低并发症的发生率，促进危重患者病情缓解和早期康复[39]–[41]。
